# Computational characterization of the behavior of a saliva droplet in a social environment

**DOI:** 10.1038/s41598-022-10180-5

**Published:** 2022-04-18

**Authors:** Ainara Ugarte-Anero, Unai Fernandez-Gamiz, Koldo Portal-Porras, Ekaitz Zulueta, Oskar Urbina-Garcia

**Affiliations:** 1 Nuclear Engineering and Fluid Mechanics Department, University of the Basque Country, UPV/EHU, Nieves Cano 12, 01006 Vitoria-Gasteiz, Araba Spain; 2 System Engineering and Automation Control Department, University of the Basque Country, UPV/EHU, Nieves Cano 12, 01006 Vitoria-Gasteiz, Araba Spain

**Keywords:** Engineering, Aerospace engineering, Biomedical engineering, Chemical engineering, Civil engineering, Electrical and electronic engineering, Energy infrastructure, Mechanical engineering

## Abstract

The conduct of respiratory droplets is the basis of the study to reduce the spread of a virus in society. The pandemic suffered in early 2020 due to COVID-19 shows the lack of research on the evaporation and fate of droplets exhaled in the environment. The current study, attempts to provide solution through computational fluid dynamics techniques based on a multiphase state with the help of Eulerian–Lagrangian techniques to the activity of respiratory droplets. A numerical study has shown how the behavior of droplets of pure water exhaled in the environment after a sneeze or cough have a dynamic equal to the experimental curve of Wells. The droplets of saliva have been introduced as a saline solution. Considering the mass transferred and the turbulence created, the results has showed that the ambient temperature and relative humidity are parameters that significantly affect the evaporation process, and therefore to the fate. Evaporation time tends to be of a higher value when the temperature affecting the environment is lower. With constant parameters of particle diameter and ambient temperature, an increase in relative humidity increases the evaporation time. A larger particle diameter is consequently transported at a greater distance, since the opposite force it affects is the weight. Finally, a neural network-based model is presented to predict particle evaporation time.

## Introduction

After experiencing the biggest pandemic in the world, so far, of the twenty-first century, experts have focused on the investigation of the consequences of living this situation, both, economically and sanitarily. The state lived during these two years ago, has left a society very different from the one known before this phenomenon. Two years of uncertainty have developed several measures to combat this virus. The first measure was quarantine for approximately two months. Once on the street, the use of masks was forced. Different types of masks were designed. Notably, mathematical studies such as Ugarte-Anero et al.^1^ demonstrate through a Computational Fluid Dynamics (CFD) research that the face-shields, which cover the entire surface of the face, do not fully protect a person from sneezing. Later, and currently is developing, the invention of different vaccines. Although different vaccines designed by large pharmaceutical companies are known, the World Health Organization (WHO) differentiates between 3 types; those that use a virus or a whole bacterium, those that use fragments that introduce an immune system response and those that use only genetic material. But the situation got worse. As expected, the virus mutated, creating new variants. Variant B.1.617.2 (delta) identified in India and variant B.1.1.7 (Alpha) identified in England, show different behavior to vaccine efficacy, according to Bernal et al.^2^. Although, after several tests, between these two types of variants, a slight difference in effectiveness is observed when the two doses are given, on the other hand, there is a greater difference when only one dose has been applied. The study by Su et al.^3^ reports that protein S is the best antigen that should be included in vaccines against COVID-19 to achieve an effective and safe.

This disease has given a boost to the field of research that drives aerosol studies. It has been researched with the aim of providing a solution to the famous SARS-CoV-2, but all these studies are based on explaining the functioning of the exhaled air to the environment, which is the route of contagion of all diseases, either by major droplets or by aerosols, according to Leung et al.^4^. The human body exhales particles between 10 µm and 100 µm of diameter in a sneeze, according to the numerical study of Chillón et al.^4^, but it is not considered aerosol until the particle reaches 5 µm of diameter, Chatterjee et al.^5^. When a person coughs, speaks, or sneezes, exhales to the environment particles with great contagion power. These particles are formed of saliva and epithelial lining fluid (ELF), Pöhlker^6^. Saliva can vary its composition depending on the person, according to Almeida PDV et al.^7^. Even if you focus on the same person, depending on what you eat during that day, it can vary the composition of saliva. Composed of 99% of water and 1% composed of other elements, notably sodium and chlorine for being more abundant, Almeida PDV et al.^7^, CFD studies make an approximation to saliva as a saline solution of 0.9 w/v, Xie et al.^8^, being the most accurate approximation of evaporation time to the aerosol, Ugarte-Anero et al.^1^. Even so, droplets of saliva have the same method of evaporation as a droplet of pure water. According to Bozic et al.^9^ , the droplet gradually evaporates over time, reducing its size. In this first stage it gets rid of water (H_2_O) molecules. Once that H_2_O has evaporated, the result is what is called droplet residue, which depends on non-water solutes inside the droplet. Lieber et al.^10^ indicates that the so-called droplet residue will remain in the environment for hours and will have a size that is 20% of the initial size of the particle studied. The latter result is what differentiates it from the evaporation mechanism of a droplet of pure water, becoming aerosol. The evaporation rate of pure water is higher than the evaporation rate of a drop of saline solution by the high solubility that NaCl has, affecting the vapor pressure of saturated water, Shahidzadeh et al.^11^. This phenomenon is marked by various characteristics that occur in the environment, such as, for example, relative humidity (RH) and ambient temperature among others, Ugarte-Anero et al.^1^, Wang et al.^12^ and Sen et al.^13^. Gregson et al.^14^ indicates two important factors in the evaporation rate of an aqueous sodium droplet, the temperature of the gaseous phase and the solute concentration in the starting droplet, deriving that a higher concentration of solute indicates a longer evaporation rate time. Stiti et al.^15^ shows that a droplet of an initial diameter of 21 µm in 2 s could become an aerosol when a temperature above 20 °C and an RH below 80% occurs. Studies such as Xie et al.^8^ , Liu et al.^16^ and Ugarte-Anero et al.^1^ state that at higher relative humidity the evaporation time of these is longer. These same studies, observe that the larger diameter of size the evaporation time is greater. The study of Xie et al.^8^ shows us that a particle of pure water at 33 °C in a space at a temperature of 18 °C and a 0% RH evaporates in 2 s when the initial diameter is 50 µm, instead, with a diameter of 100 µm the time amounts to 7.2 s. As the size increases the evaporation time increases. According with the study of Shahidzadeh et al.^11^ the evaporation rate increases linearly with the diameter of the initial droplet. If the focus is shifted to investigate the reaction of these droplets to ambient temperature, the investigation of Wang et al.^12^ shows that the lifetime of a droplet in wet spaces is less when the temperature decreases. In dry environments, on the other hand, the shelf life of the droplet is longer when the temperature decreases. He also points out that average particles are very sensitive to relative humidity, and it might be different to study their precise behavior.

Evaporation is important to study how long it takes to become aerosol and achieves a strong contagion capacity. Although, once you have that particle less than 5 µm in diameter, you need to know where it’s going. This phenomenon depends on many factors; the speed at which it is generated, whether we are in a closed or open place, anyhow there is a high-speed wind or a soft breeze, regardless there is ventilation, etc. In fact, the study by Cravero et al.^17^, indicates that with adequate ventilation in an indoor environment completely changes the direction of exhaled flow. Stiti^15^ has found that a particular 80 µm becomes solid waste before reaching the ground when the person exhaling it has a height of 1.6 m from the mouth to the ground. CFD studies such as Dbouk et al.^18^ and Li et al.^19^ warn that with an external wind greater than 1.1 m/s can reach up to 6 m away. This current, if it is descending, can remove the infected droplets in just 10 s, on the contrary, an ascending current helps to have a viral load of 50% around the height of people, according to de Oliveira^20^. Consequently, in a closed space, such as the study by Chillon et al.^4^ the particles do not exceed one and a half meters of distance. The study by Zhao et al.^21^ combines fate with RH and ambient temperature and ensures that in humid environments and low temperature droplets travel a greater space. In dry environments and high temperature, the number of aerosols is higher, underline what previously cited by the studies of Xie et al.^8^ and Wang et al.^12^ that in dry space the evaporation is slower.

The aim of this work is to study through computational simulations the behavior of a single saliva particle exposed to different environmental characteristics in a social environment. Launch the particle, without velocity, from a height of 1.6 m and study in a first case its evaporation time at 0 °C, 14 °C and 35 °C in a relative humidity range of 0% to 80%. The diameter of the particle are 25 µm, 50 µm, 75 µm and 100 µm. Finally, the fate of droplets saliva (10–90 µm) is studied at a temperature of 14 °C and 35 °C and relative humidity of 0%, 20%, 40%, 60% and 80%, with a velocity of 0 m/s, affecting only the gravity and weight of the droplet of saliva.

## Materials and methods

### Computational domain and initial conditions

**Figure 1:**
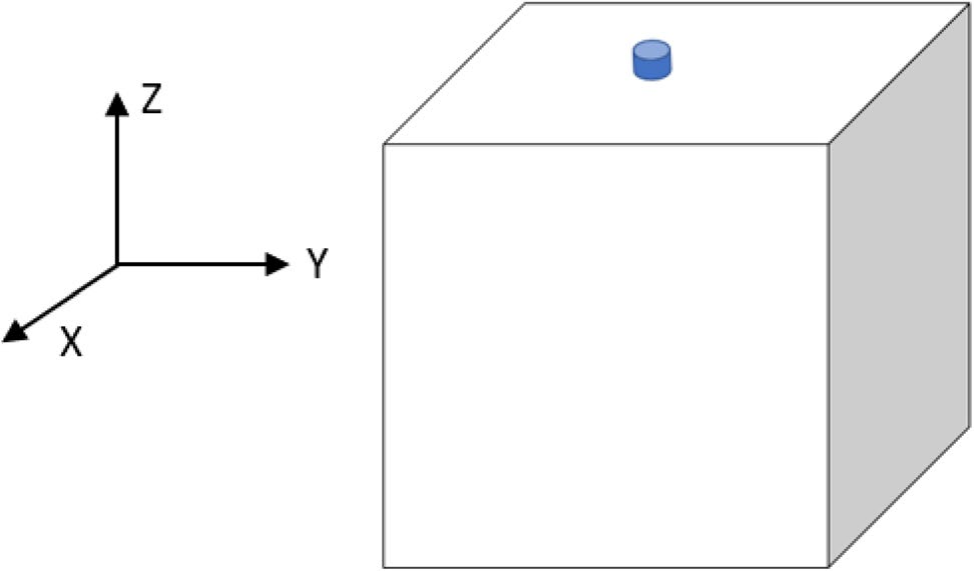
Geometry of the domain. It consists of a box with measures of 2 m × 2 m × 1.6 m (X, Y, Z). At a height of 1 m × 1 m × 1.6 m a cone injector is collapsing simulating the particle.

In order to analyze through CFD techniques the most remarkable properties of a droplet of saliva, the domain used has been a box of 2 m X 2 m X 1.6 m (X, Y, Z). The generated computational domain, the cube, composed of a single boundary, taken as a wall. The six walls act as a barrier to stop the particle getting injected there. Enclosed space, study based on an indoor environment, without air or external wind affecting the particle. It should be noted that the value of Z is equal to 1.6 m for the average height that can take the mouth of a human. Figure 1 shows the bucket with the cone injector, placed in the central part of the upper face, specifically [1, 1, 1.6]. This injector launches a particle into outer space simulating a human cough. Instead of generating a particle size distribution, a particular particle will be studied, with a specific diameter the importance it has when exhaled. The injector will make as if it were a human mouth when generating a sneeze.

**Figure 2:**
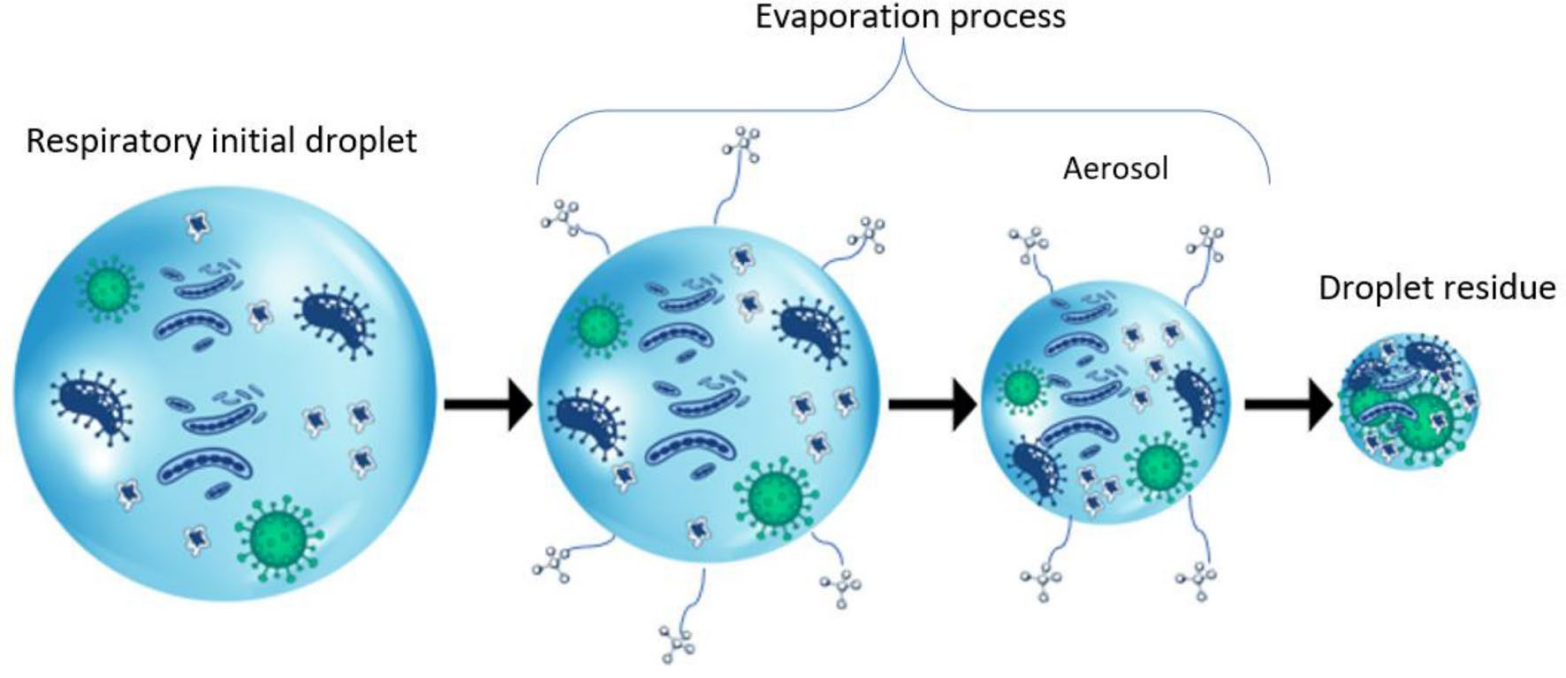
Evaporation process of a droplet of saliva. Once the particle is exhaled into the environment, the evaporation process begins by evaporating saliva water. When it reaches 5 µm of diameter it becomes aerosol, becoming droplet residue.

First, taking as a reference the temperatures reached by European countries, the pure water particle has been subjected to 3 different temperatures: 0 °C, 14 °C and 35 °C. In contrast, relative humidity varies from 0 to 80%. The diameter of the particle takes values of 25 µm, 50 µm 75 µm and 100 µm at 36 °C. According to the study of Almeida PDV et al.^7^, saliva is composed of 99% water and the rest is formed by proteins, enzymes and electrolytes, among others. Also, the electrolytes, sodium and chloride stand out. Research by Xie et al.^8^ models saliva as a saline solution with a concentration of 150 mM of ions and cations, affecting only saturation pressure. In Fig. 2, you can see the process of evaporation of an exhaled droplet into the human environment. The current work has the limitation of not being able to study aerosol behavior. The process of complete evaporation of a droplet of saliva has been given as a droplet of pure water, without reaching the residue of the droplet.

**Figure 3:**
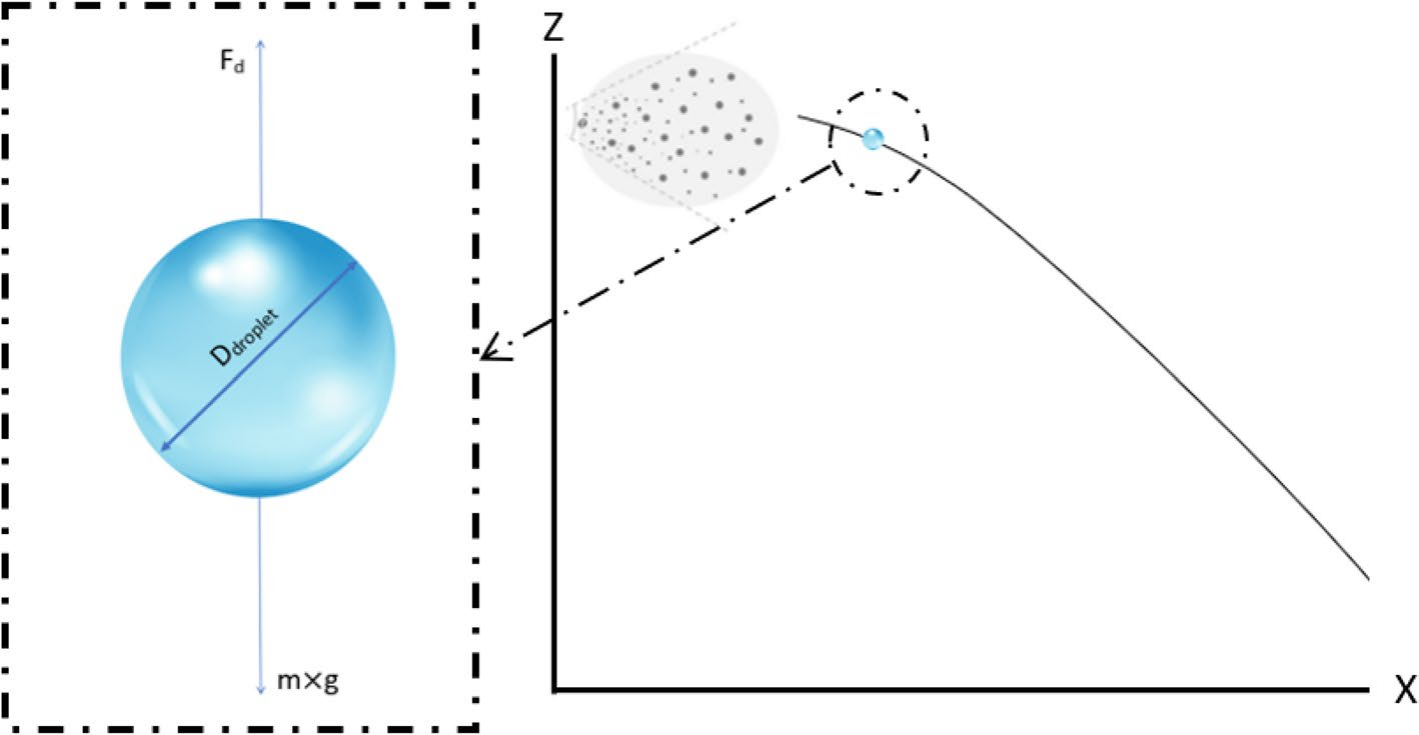
Forces that affect a droplet of saliva. Droplet falling freely affected by gravity, whether weight, and drag force.

In the other hand, the behavior of the saliva particle in relation to the distance travelled has been studied. For this case, the temperatures are 14 °C and 35 °C and relative humidity of 0%, 20%, 40%, 60% and 80%, with a starting speed of 0 m/s, so it has only affected the forces of gravity, the weight of the drop, according to Bozic^9^. According to the CFD model of Pendar et al.^22^, approximately the experimental average speed that can catch a sneeze is 20 m/s, but when it is emitted to the environment in just a few seconds loses the speed adapting to that of the environment, Ugarte-Anero et al.^1^. Figure 3 shows a sketch of the forces to which a particle is subjected.

**Figure 4:**
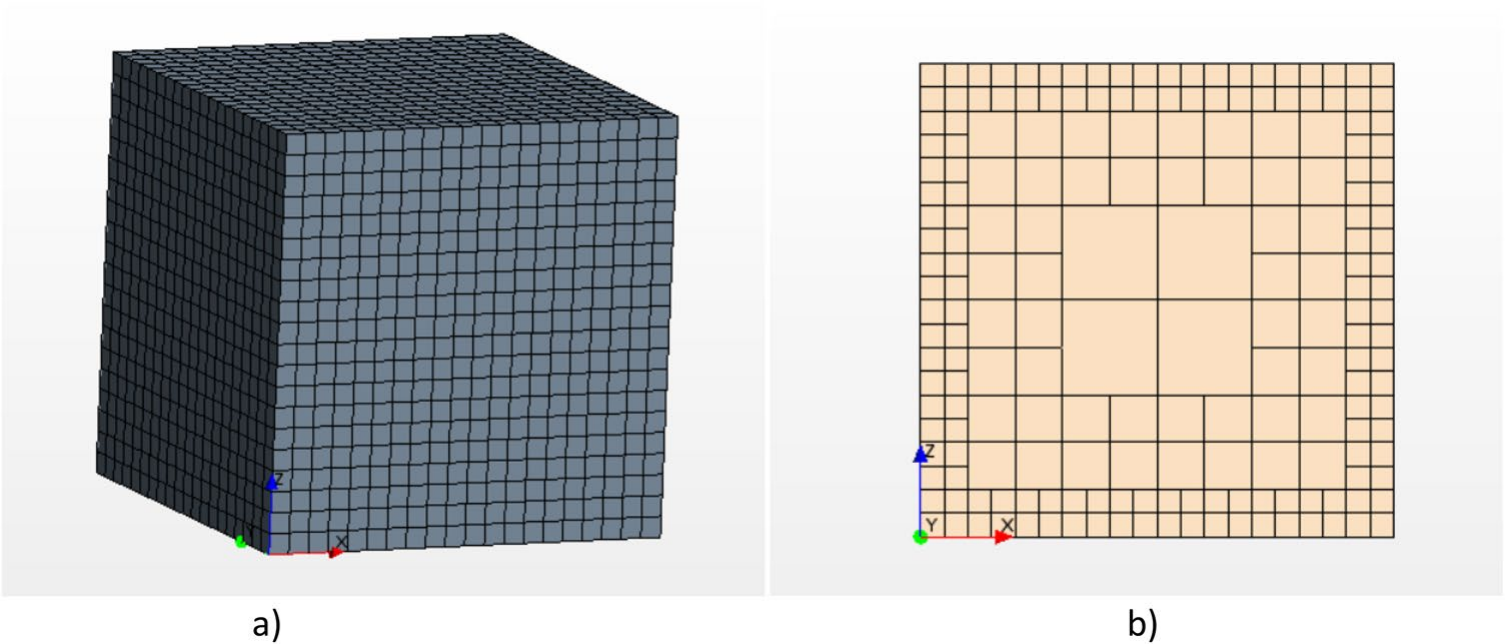
Mesh distribution. (**a**) Geometry of the mesh cube; (**b**) plane through which the particle passes.

A generalized Richardson extrapolation method^23,24^ was performed to achieve the mesh independency study. This method consists of estimating the value of the analyzed parameter when the cell quantity tends to infinite from a minimum of three meshes. In the current study, a coarse mesh (1176 cells), a medium mesh (2528 cells), and a fine mesh (4360 cells) were considered. Figure 4a shows the meshed domain and Fig. 4b illustrates the plane through which the particle falls down. Appendix A shows a complete description of the mesh dependency study carried in the present work. The estimated values (RE) of the evaluated parameters are close to the ones obtained with the fine mesh for all cases. Note that a monotonic convergence is achieved since R values are positive and less than one. A mesh refinement ratio of approximately r = 2 has been chosen and p is the order-of-accuracy, see Stern et al.^25^. Tables A1 and A2 show the results of the Richardson Extrapolation based method.

## Numerical set up

The study is solved as a two-phase flow situation, introducing the two-way coupling module. The droplet of pure water has form the Lagrangian phase. The particle, in question, has an initial temperature of 36 °C but then will go down, taking the temperature of the external environment, according to the statement offered by Redow^26^. Saturation pressure follows Antoine equation, when the particle is pure water. The latter characteristic varies when it comes to simulating saliva. A saline solution of 0.9% w/v, have a saturation pressure in accordance with Raoult’s Law, as indicated by Xie^8^.

1 $${\text{P}}_{{{\text{va}},{\text{s}}}} = {\text{X}}_{{\text{d}}} {\text{P}}_{{{\text{va}}}} \left( {{\text{T}}_{{\text{w}}} } \right)$$

where $P_{va,s}$ is the saturation pressure of the droplet in the saline mixture, $P_{va}$ is the saturation pressure indicated by the Antoine equation at an indicated temperature (in this case at 36 °C) $\left( {T_{w} } \right){ }$ and $X_{d}$ is the mole fraction of the droplet, that is calculated as shown in Eq. (2).

2 $$X_{d} = \left( {1 + \frac{{6i{\text{m}}_{{\text{s}}} {\text{M}}_{{\text{w}}} }}{{\pi \rho_{{\text{L}}} {\text{M}}_{{\text{s}}} ({\text{d}}_{{\text{p}}} )^{3} }}} \right)^{ - 1}$$

where m_s_ the mass of solute in the droplet; $d_{p}$ is the diameter of the droplet studied; Mw is the molecular weight of water and Ms is the molecular weight of solute, the ion factor “i” is equal to 2.

The Reynolds-averaged Navier–Stokes (RANS) equations with k-ω Shear Stress Transport (SST) turbulence model, developed by Menter et al.^27^ have been introduced in this work. The UpWind algorithm was employed for the pressure–velocity coupling and a linear upwind second order scheme was used to discretize the mesh. Figure 3 shows the forces to which the droplet is subjected, which is initially assumed to be spherical. The influence of the gravity force was taken into account. The Taylor analogy breakup (TAB) model was implemented to provide a solution to particle distortion and break up. Also, the turbulent particle dispersion with the exact eddy interaction time is taken into account. The drag force takes the value according to Schiller-Naumann mathematics model. Model used in the numerical study of Wang et al.^12^ and in the investigation of de Oliveira et al.^20^. The model simulated the drag between the two current phases. Equation 3 shows the expression to calculate the drag coefficient Cd.

3 $${C_d}\left\{ \begin{array}{l} \frac{{24}}{{Re}},\quad \quad Re \le 1\\ \frac{{24}}{{Re}}\left( {1 + 0.15R{e^{0.687}}} \right),\\ 0.44,\quad \quad \;Re > 1000 \end{array} \right.\;\;\;1 < Re \le 1000$$

where Re is the Reynolds number.

Once the respiratory droplets are exhaled into the social environment where humans are found, the process of evaporation begins. For this, Busco et al.^28^ introduces the quasi-steady evaporation model, incorporated in this project. Formula 4 shows the equation in which the model is governed, subject to mass loss.

4 $$\dot{m}_{p} = g^{*} \times A_{s} ln\left( {1 + B} \right)$$

where $g^{*}$ is the mass transfer conductance and $A_{s}$ is the droplet surface area. B is the Spalding transfer number. $g^{*}$ and B are defined as:

5 $$g^{*} = - \left( {\frac{{\rho_{p} D_{v} Sh}}{{D_{p} }}} \right)$$

6 $$B = \frac{{Y_{i,s} - Y_{i,\infty } }}{{1 - Y_{i,s} }}$$

In the Eqs. (5 and 6), $\rho_{{\text{p}}}$ is the density of the particle liquid phase, ${\mathrm{D}}_{\mathrm{v}}$ and ${\mathrm{D}}_{\mathrm{p}}$ are the molecular diffusivity of the vapor phase and of the liquid phase, respectively. Sh is the correlation for de Sherwood number. ${\mathrm{Y}}_{\mathrm{i},\mathrm{s}}$ is the vapor mass fraction at the surface and ${\mathrm{Y}}_{\mathrm{i},\infty }$ is the vapor mass fraction inside the fluid phase.

The second important part of this research is the simulation of the atmosphere. The cube has simulated this and have been solved by equations for the continuous phase expressed in Eulerian form. Non-reactive species that after determining their properties, the total binding property is calculated as a mass function of the components of the mixture. Busco et al.^28^ incorporates Eq. (7), based on the mass-weighted mixture method.

7 $${{\upphi}_\text{mix}} = \mathop \sum \limits_{\text{i} = 1}^{\text{N} = 2} {{\upphi} _\text{i}}{\text{Y}_\text{i}}$$

where Yi is the mass fraction of air and water vapor and фi is the property values of mixture component. N is the total number of components in the mixture.

Composed of dry air and water vapor, varying their mass composition gives rise to relative humidity. By observing the Kukkonen et al.^29^ appendix the density and viscosity of the water vapor air and liquid water have been taken as the value marked by the equations of that report when changing temperature. In the current work, the commercial CFD code STAR-CCM + v.14.02 (Siemens, London, UK) was used to define and solve the numerical model of aerosols. A personal server-clustered parallel computer with Intel Xeon © E5-2609 v2 CPU @ 2.5 GHz (16 cores) and 45 GB RAM were used to run all the simulations.

### Validation

In the case of computational simulations, validation with experimental results is required. The current work reports on the evaporation of pure water droplets and on the fate of saliva droplets, therefore, has validated both, evaporation and distance.

**Figure 5:**
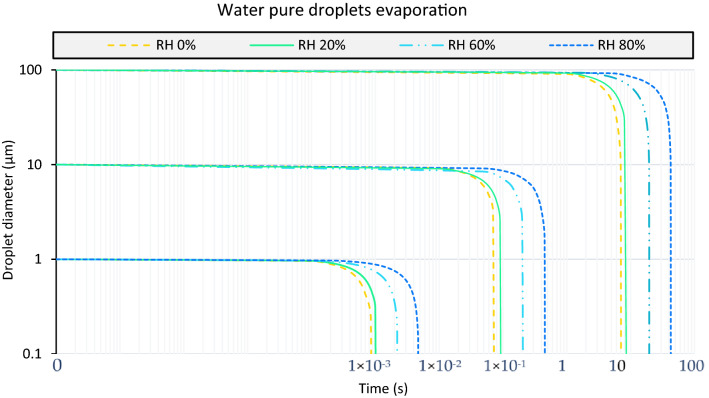
Water pure droplets evaporation. Particle diameter of 1 µm, 10 µm and 100 µm at a temperature of 310.15 K. Simulated in an environment with variable relative humidity of 0%, 20%, 60% and 80% at a constant temperature of 293.15 K.

The same method used by Redow^26^, Mowraska^30^, Li^19^ and Ugarte-Anero^1^ was used for the validation of evaporation. A study in which different diameters of droplets of pure water (1 µm 10 µm and 100 µm) with a temperature of 310.15 K are subjected to an environment of 293.15 K and RH varies to see the evaporation time. The results obtained are similar to those shown by the aforementioned works, shown in Fig. 5.

**Figure 6:**
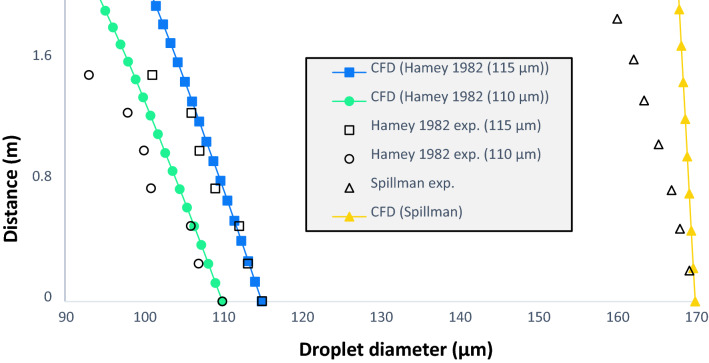
Water pure droplets fate. Droplets of 110 µm and 115 µm fall freely at an initial temperature of 289 K in an environment of 293 K to an RH of 70%, called as Hamey study. Spillman study with the same purpose as the above, of a particle of 170 µm of diameter at a temperature of 25 °C in an environment of 31 °C temperature and HR of 68%.

Following the same philosophy, a pure water droplet and a saliva droplet only have difference in the vapor pressure in this study, therefore, affect the same forces and we can go ahead with studies like Hamey et al.^31^ and Spillman et al.^32^ that show the path of a droplet of pure water. Hamey study consists of two droplets of water, one of 110 µm and another of 115 µm of diameter, at a temperature of 289 K in an environment at 293 K and relative humidity of 70% and observe its path having let the droplet fall freely. With the same objective, the Spillman studio launches a 170 µm of diameter particle at 25 °C in a 31 °C environment with a relative humidity of 68%. Figures 5 and 6 show the results obtained by comparing our CFD data with the experimental studies. Instead, Table 1 shows the error as a percentage between the experimental data of the Hamey and Spillman and the results obtained with CFD techniques.

**Table 1:** Calculated error between the results obtained by CFDs and the experimental data of the Hamey and Spillman studies.

Hamey 1982 110 µm	Hamey 1982 115 µm	Spillman
0.000301%	0.034%	0.161%
1.160%	0.019%	0.287%
0.393%	0.554%	0.852%
3.615%	0.597%	1.348%
2.693%	1.645%	2.201%
2.955%	0.932%	3.114%
6.379%	4.083%	3.801%
3.532%		4.995%

## Results

In a sneeze, a particle distribution of 10–100 µm diameter is generated. The average diameters selected to study the case are: 25 µm, 50 µm, 75 µm and 100 µm. The human body is at a temperature of approximately 36 °C, so the exhaled particles have an initial temperature of 36 °C. With a minimum temperature of 0 °C, a particle of 100 µm of diameter, in an environment with relative humidity of 80%, reaches an evaporation time of 61.2 s. In contrast, with a temperature of 14 °C and constant RH the evaporation time is equal to 54.4 s. And, with 35 °C, and the same characteristics, 30.3 s is the time it takes for that particle to evaporate.

**Figure 7:**
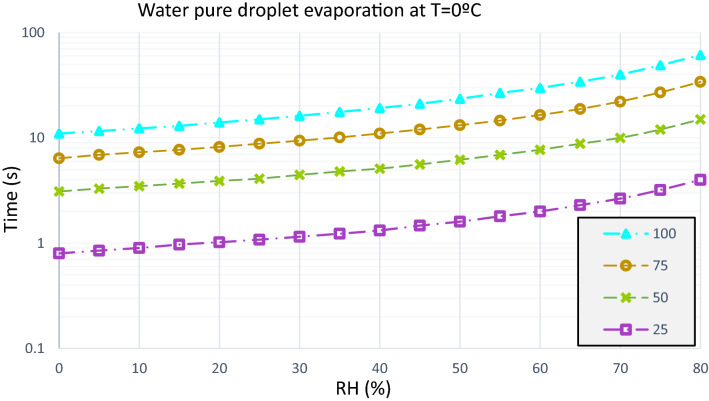
Water pure droplets evaporation. Time elapsed in the evaporation process of a droplet of pure water at an ambient temperature of 0 °C, with variable relative humidity and particle diameter of 25 µm, 50 µm, 75 µm and 100 µm.

**Figure 8:**
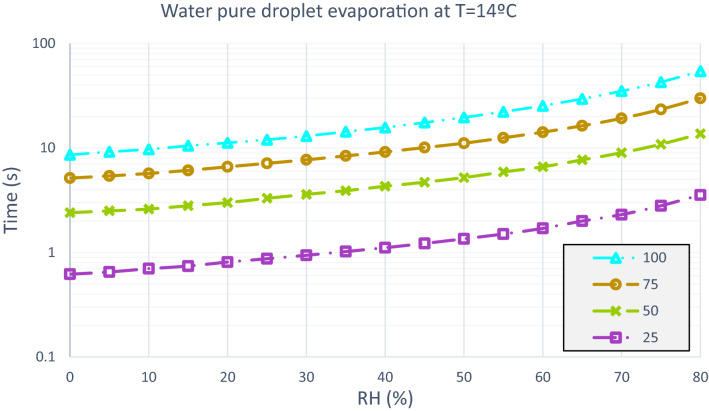
Water pure droplets evaporation. Time elapsed in the evaporation process of a droplet of pure water at an ambient temperature of 14 °C, with variable relative humidity and particle diameter of 25 µm, 50 µm, 75 µm and 100 µm.

**Figure 9:**
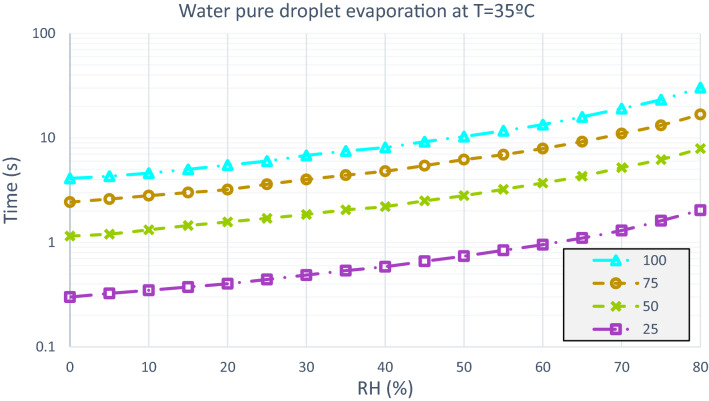
Water pure droplets evaporation. Time elapsed in the evaporation process of a droplet of pure water at an ambient temperature of 35 °C, with variable relative humidity and particle diameter of 25 µm, 50 µm, 75 µm and 100 µm.

When it is a 50 µm particle with a temperature of 14 °C, in an environment with a relative humidity of 50% the evaporation time is 5,2 s and RH 80% the evaporation time is equal to 13.7 s. The distribution of evaporation of the test droplets is analyzed in Figs. 7, 8 and 9.

**Figure 10:**
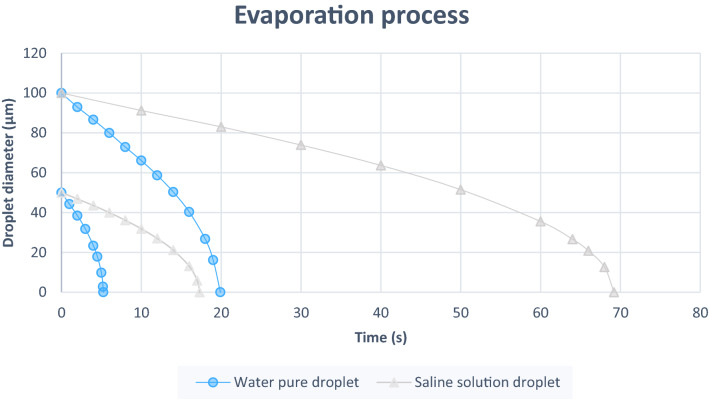
Difference of evaporation process. Under conditions of relative humidity of 50% and ambient temperature of 14 °C. A particle of pure water with 50 µm of diameter evaporates 17 s before one of saline solution droplet. However, a particle of pure water with 100 µm of diameter evaporates 50 s before one of saline solution droplet.

By converting saliva into a saline solution, the evaporation time of saline solution droplet is 17 s approximately more in comparison with pure water droplet. Both cases were performed under the conditions of a RH = 50% and an environment temperature of 14 °C, and the diameters of the droplet was 50 µm. Instead, as the diameter of the saline particle increases this difference is more notary. A particle of pure water with 100 µm of diameter evaporates 50 s before one of saline solution droplet. Figure 10 shows the difference in the evaporation process between a droplet of pure water and a droplet of saline solution of a 50 µm and 100 µm droplet.

In the case of studying the distance that the droplets generated in a sneeze or cough can travel, the law of gravity is kept, the more diameter the more weight, the longer distance traveled. An average particle of 50 µm, at a relative humidity of 60% travels 0.24 m, before evaporating. Rather, at a humidity of 20% the particle stays at a distance of 1.5 m from the ground, all in an environment with a temperature of 14 °C. With the same peculiarities, when RH = 60%, a particle of 90 µm has travelled the 1.6 m before evaporating and when the RH = 20% the distance traveled has been 0.95 m.

**Figure 11:**
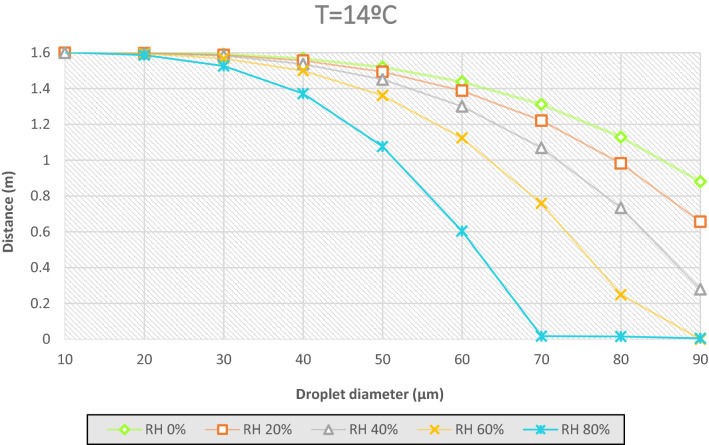
Distance covered by droplets of saliva (10 µm, 20 µm, 30 µm, 40 µm, 50 µm, 60 µm, 70 µm, 80 µm and 90 µm) from a height of 1.6 m at a temperature of 14 °C and with a variable relative humidity.

**Figure 12:**
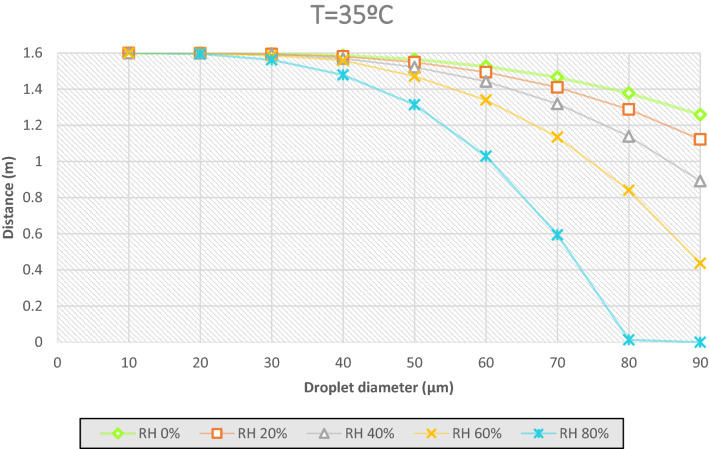
Distance covered by droplets of saliva (10 µm, 20 µm, 30 µm, 40 µm, 50 µm, 60 µm, 70 µm, 80 µm and 90 µm) from a height of 1.6 m at a temperature of 35 °C and with a variable relative humidity.

Considering Figs. 7, 8 and 9, the effect of the evaporation process, a 50 µm of diameter particle at ambient temperature of 35 °C and a relative humidity of 60% passes through a distance of 0.13 m before evaporating, from a height of 1.6 m. Figures 11 and 12 show the fate of the particles released from a height of 1.6 m from the ground, corresponding to the average height of the mouth of an adult person. The heavier droplets manage to reach the soil instead, the smaller droplets evaporate into the air leaving the droplet residue in the environment. The droplet residue generated by the larger droplets would settle on the surface.

**Table 2:** Differences between the time of sedimentation and the time of evaporation.

Particle diameter	Time of sedimentation (s)	Time of evaporation (s)
25 µm	85.4	0.74
50 µm	21.3	2.8
75 µm	9.5	6.2
100 µm	5.3	10.3

Time of sedimentation calculated with empirical formulas and time of evaporation taken out with this study CFD.

Then, according with the results obtained by studying the particle dynamics, the results of the time of thirst determined by empirical formulas is studied. Equation 8, provided by the study of Bozic et al.^9^ ,indicates the parameters that influence the sedimentation time. Table 2 shows the results obtained from four examples at a relative humidity of 50%. It should be noted that a particle with a diameter of 80 µm in an environment with a temperature of 35 °C and RH = 80%, can be transported from a height of 1.6 m, before evaporation, at a distance of 0.015 m from the ground over a time of 7.9 s. With the empirical formula we obtain that the sedimentation time of that droplet is 0.2 s more than the evaporation time, time that would elapse to travel the distance of 0.015 m previously mentioned. Only the 100 μm particle has been evaporate after deposition in the soil, the others have evaporated in the air before reaching the marked distance.

8 $$t_{sedimentation} = {\text{h}} \div ({\xi } \times \left( {R_{droplet} )^{2} } \right)$$

where h = 1.6 m, in this case since it is the average height of the human mouth, R_droplet_ is the radius of the particle and the letter ξ equals to the number $1.2 \times 10^{8}$ m^−1^ s^−1^.

## ANN-based evaporation time prediction model

Aiming to obtain the evaporation time of the particles, an Artificial Neural Network (ANN) is developed. Deep Learning techniques, which include neural networks, are exceptional tools for modelling a wide variety of systems due to their properties and advantages in comparison with other traditional techniques, see Lopez-Guede et al.^33,34^. Among these advantages and properties, the most remarkable ones are their ability to learn and their fast computational speed.

In the present paper a multi-layer model with two hidden layers is used. The evaporation time of the particle is calculated by Eq. (9), and the outputs of each neuron of the hidden layers follow a sigmoid function, which is defined in Eq. (10). The ANN with these parameters represents a typical Multilayer Perceptron with Backpropagation (BP-MLP) configuration. The postsynaptic $h_{i}$ of each $i$ neuron is calculated using a linear combination defined in Eq. (11).

9 $${\text{Time}} = \mathop \sum \limits_{i = 1}^{{i = N_{hidden} }} \omega_{i} \cdot g_{i} \left( {\vec{x}} \right) + \theta$$

10 $$g_{i} \left( {\vec{x}} \right) = \frac{1}{{1 + e^{{ - h_{i} }} }}$$

11 $${h_i}\left( {\vec x} \right) = \mathop \sum \limits_{j = 1}^{j = {N_{inputs}}} {\omega^\prime_{i,j}}\cdot{x_j} + {\theta^\prime_i}~$$

where $\omega_{i}$ represents the weights of the output layers and ${\omega }_{ij}$ the weights of the input hidden layers.

**Figure 13:**
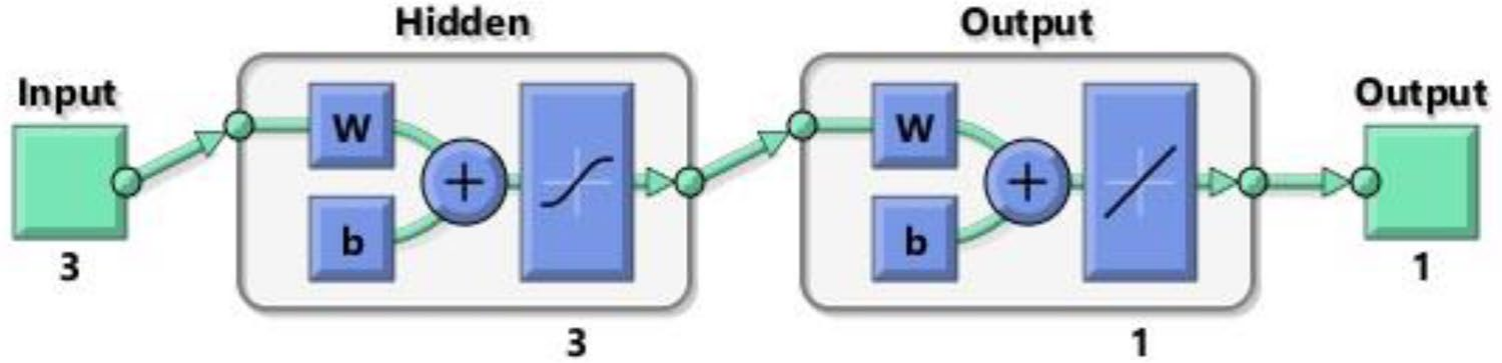
Sketch of the designed ANN.

The designed network has three different inputs, temperature, particle diameter and relative humidity: and a single output, the evaporation time. As mentioned before, it has two hidden layers. A schematic view of the ANN is provided in Fig. 13.

For training the network, the data has been separated into 70% training, 20% validation and 10% test. Matrices 12–15 contain the values of the input hidden layer weights (${\omega }_{ij}$), output layer weights (${\omega }_{i}$), hidden layer threshold parameters ($\sigma$) and output layer threshold parameters ($\sigma ^{\prime}$). These matrices have been obtained in the training process of the network and are fundamental for a correct prediction of the evaporation time.

12 $${\upomega }_{{{\text{ij}}}} = \left[ {\begin{array}{*{20}c} { - 0.279} & {0.1721} & {0.3654} \\ {0.4295} & { - 0.5551} & { - 1.5249} \\ { - 0.2749} & {0.1648} & {0.3602} \\ \end{array} } \right]$$

13 $${\upomega }_{{\text{i}}} = \left[ {\begin{array}{*{20}c} {0.3913} & { - 0.0049} & { - 1.0907} \\ \end{array} } \right]$$

14 $${\upsigma } = \left[ {\begin{array}{*{20}c} { - 1.9907} \\ {3.0613} \\ { - 2.5123} \\ \end{array} } \right]$$

15 $${{\sigma^{\prime}}} = \left[ { - 695.3689} \right]$$

**Figure 14:**
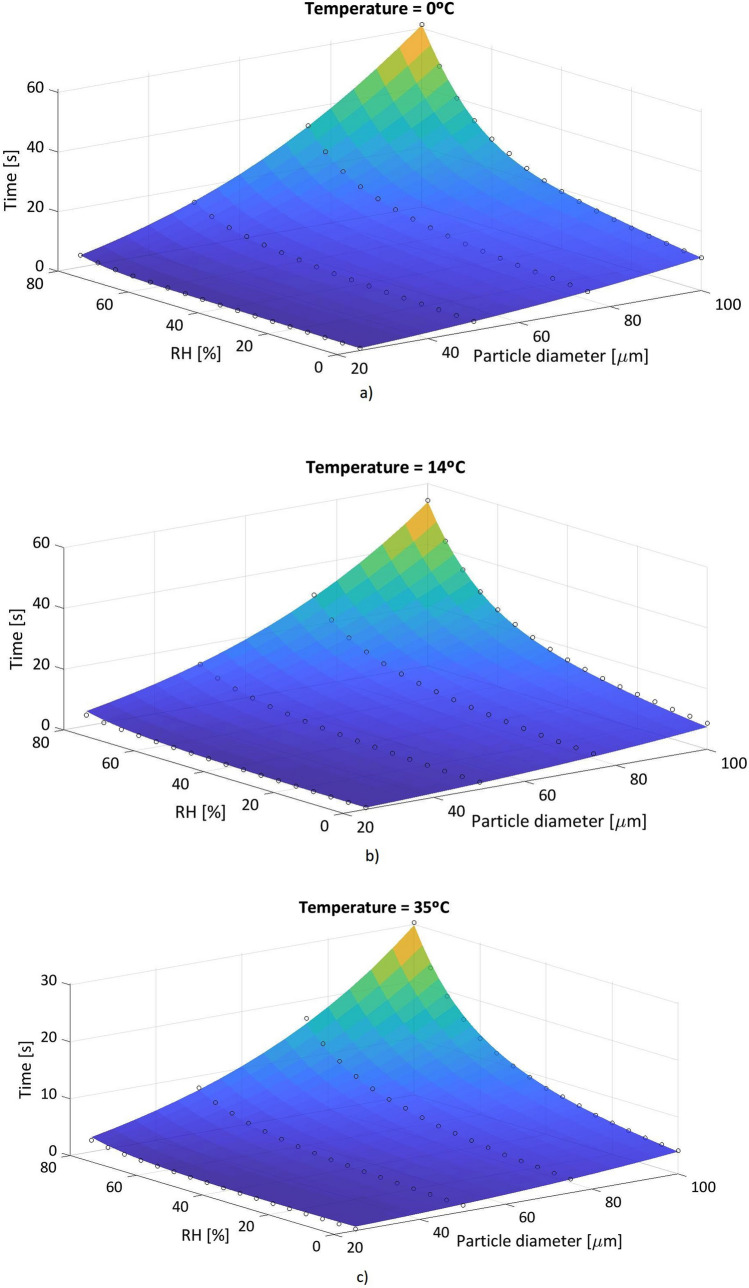
Comparison of the evaporation times of the ANN predictions (colored surface) and CFD results (black circles). (**a**) T = 0 °C; (**b**) T = 14 °C; (**c**) T = 35 °C.

With the obtained network, predictions of evaporation times under conditions different from those of CFD simulations are made. Figure 14 shows the results obtained for 0 °C Fig. 14a, 14 °C Fig. 14b and 35 °C Fig. 14c.

**Figure 15:**
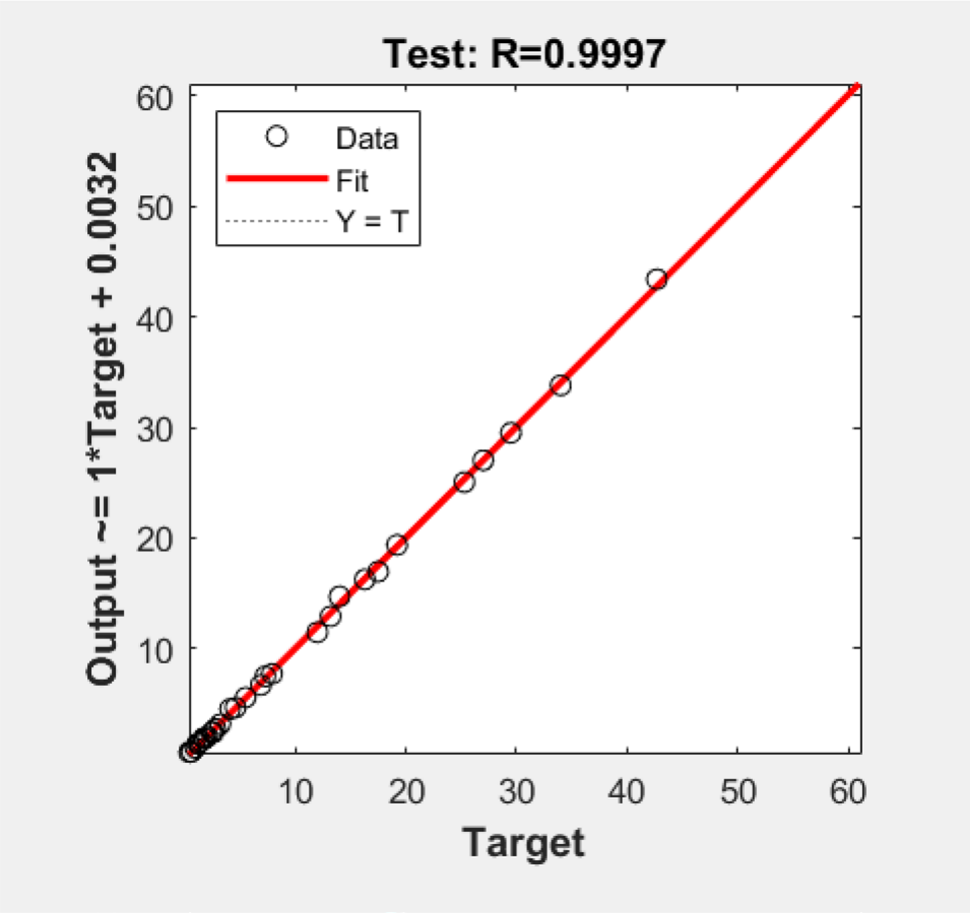
ANN training correlation coefficients. The vertical axis shows the ANN predictions and the horizontal axis the CFD data.

To demonstrate the accuracy of the network the correlation coefficient (R-value) of the predictions in the cases corresponding to the test-set mentioned above is analyzed, as shown in Fig. 15. This coefficient quantifies the relation between the ground-truth values and the predicted values. Therefore, to ensure the accuracy of the predictions this coefficient should be as close to 1 as possible. In this case, R = 0.9997, therefore, the ANN is able to correctly predict the evaporation time.

## Conclusions

In the current work, the dynamics of a single saliva droplet exposed to different characteristics has been studied by means of CFD based numerical simulations. Launch the particle, without velocity, from a height of 1.6 m, average distance from a human mouth, and study in a first case its evaporation time at 0 °C, 14 °C and 35 °C in a relative humidity range of 0% to 80%. The diameter of the particle was 25 µm, 50 µm, 75 µm and 100 µm. Finally, we study the fate of these particles at a temperature of 14 °C and 35 °C and relative humidity that varies from 0 to 80% in a range of 20, with a velocity of 0 m/s, affecting only the gravity and weight of the saline solution droplet. The diameters studied in this case have been from 10 µm to 90 µm in a range of 10.

Note that when a particle moves in an environment with temperature and RH constant, as the diameter increases the evaporation time is greater. It is also observed that by increasing the droplet diameter, the variation in temperature is more noticeable. That is, the difference in evaporation time is greater in a larger particle, once comparing two temperatures, than in a smaller one. Similar effect happens when, instead of increasing the particle diameter, the relative humidity is increased. In contrast, with a constant particle diameter and without variation on the relative humidity, when the ambient temperature is lower, then the evaporation time is larger. On the other hand, when the variable to be altered is the RH, if the value is increased, the evaporation time increases as well. Taking all this into account, we can state that the characteristics of the environment, and more specifically the ambient temperature and relative humidity, are parameters that significantly affect the evaporation process. The evaporation rate of a droplet of pure water consists of a similar evaporation system as a droplet of saline solution. However, a droplet of saliva after the water evaporates, the solid residue is reduced, which remains in the environment with strong contagion capacity. Additionally, we conclude that a larger particle diameter is consequently transported at a greater distance, since the opposite force that affects is the weight and the greater the diameter, the greater the weight.

## Supplementary Information

Supplementary Information.

## Data Availability

All data generated or analyzed during this study are included in this published article and its supplementary information files.
