# Supplementary material for: Computational characterization of the behavior of a saliva droplet in a social environment
Source: Sci Rep. 2022 Apr 18;12:6405. doi: 10.1038/s41598-022-10180-5 (PMC9016067; doi:10.1038/s41598-022-10180-5)
Supplement: Supplementary file 1 — Supplementary Information. [file 41598_2022_10180_MOESM1_ESM.pdf]

Appendix A

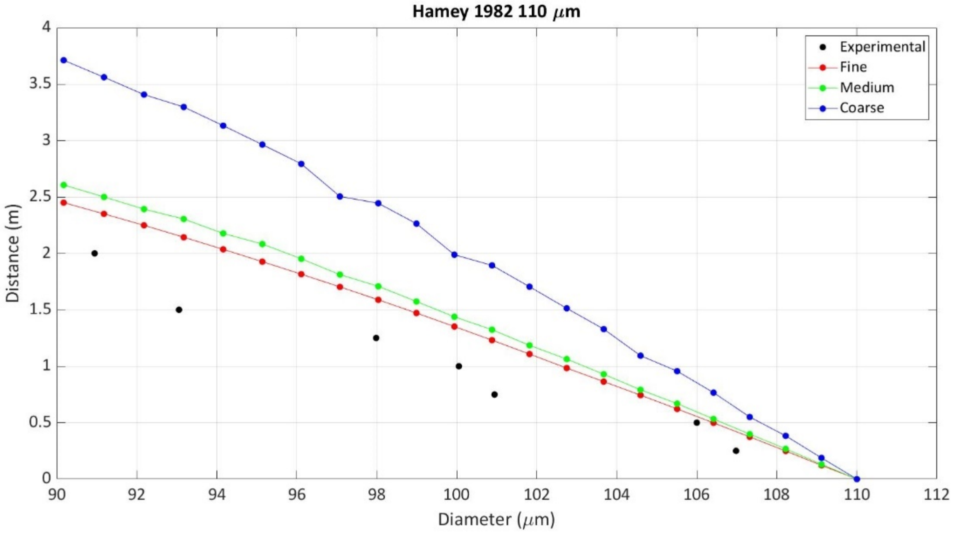

Figure A1. Water pure particle diameter vs distance achieved before evaporation for three levels of grids. Experimental data of Hamey et al. [31] with a particle of 110 µm of diameter has been used for numerical result validation.

Table A1. Results of grid convergence study based on the Richardson Extrapolation method for the case of a particle of 110 µm of diameter.

	Mesh			Richardson Extrapolation		
Diameter	Coarse	Medium	Fine	RE	p	R
109,12	0,1875	0,1304	0,1219	0,1234	2,7539	0,1482
108,22	0,3831	0,2677	0,2490	0,2526	2,6215	0,1625
107,32	0,5506	0,3983	0,3744	0,3788	2,6719	0,1569
106,42	0,7665	0,5329	0,4982	0,5043	2,7539	0,1482
105,51	0,9568	0,6687	0,6219	0,6310	2,6215	0,1625
104,59	1,0941	0,7915	0,7440	0,7528	2,6719	0,1569
103,67	1,3298	0,9294	0,8644	0,8770	2,6215	0,1625
102,75	1,5139	1,0638	0,9841	1,0013	2,4960	0,1773
101,82	1,7055	1,1856	1,1086	1,1220	2,7539	0,1482
100,88	1,8944	1,3241	1,2314	1,2494	2,6215	0,1625
99,94	1,9890	1,4388	1,3525	1,3686	2,6719	0,1569
98,99	2,2645	1,5743	1,4719	1,4898	2,7539	0,1482
98,04	2,4452	1,7090	1,5894	1,6126	2,6215	0,1625
97,08	2,5055	1,8125	1,7038	1,7240	2,6719	0,1569
96,11	2,7945	1,9532	1,8164	1,8430	2,6215	0,1625
95,14	2,9652	2,0837	1,9274	1,9611	2,4960	0,1773
94,16	3,1332	2,1782	2,0366	2,0612	2,7539	0,1482
93,17	3,2985	2,3054	2,1441	2,1754	2,6215	0,1625
92,18	3,4087	2,3934	2,2498	2,2734	2,8219	0,1414
91,18	3,5624	2,5013	2,3512	2,3759	2,8219	0,1414
90,17	3,7133	2,6072	2,4508	2,4765	2,8219	0,1414

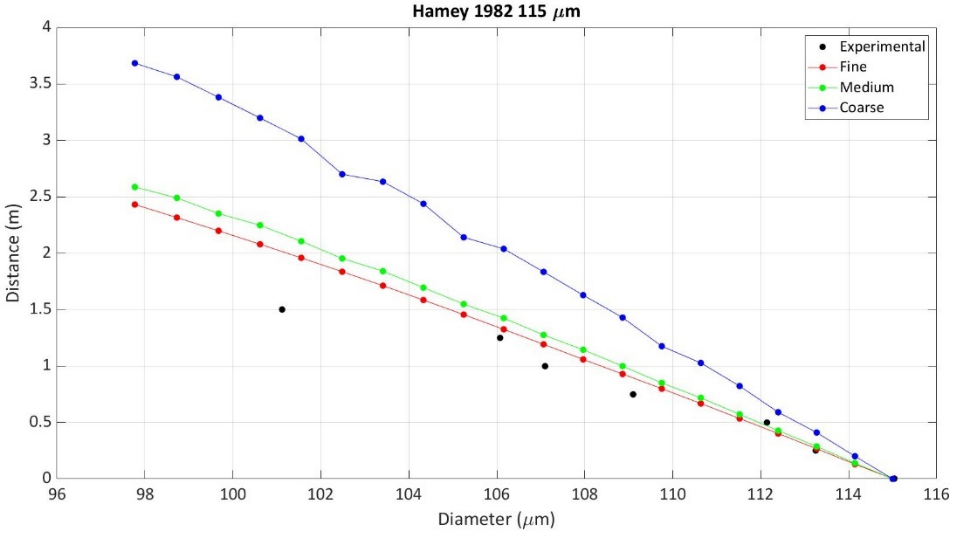

Figure A2. Water pure particle diameter vs distance achieved before evaporation for three levels of grids. Experimental data Hamey et al. [31] with a particle of 115 μm of diameter has been used for numerical result validation.

Table A2. Results of grid convergence study based on the Richardson Extrapolation method for the case of a particle of 115 μm of diameter.

		Mesh		Richardson Extrapolation		
**Diameter**	**Coarse**	**Medium**	**Fine**	**RE**	**p**	**R**
114,14	0,2005	0,1394	0,1303	0,1319	2,7539	0,1482
113,27	0,4104	0,2868	0,2667	0,2706	2,6215	0,1625
112,40	0,5905	0,4271	0,4015	0,4063	2,6719	0,1569
111,52	0,8227	0,5719	0,5348	0,5412	2,7539	0,1482
110,64	1,0275	0,7182	0,6679	0,6777	2,6215	0,1625
109,75	1,1757	0,8505	0,7994	0,8089	2,6719	0,1569
108,86	1,4298	0,9993	0,9294	0,9429	2,6215	0,1625
107,96	1,6286	1,1444	1,0586	1,0771	2,4960	0,1773
107,06	1,8349	1,2756	1,1927	1,2071	2,7539	0,1482
106,16	2,0387	1,4249	1,3251	1,3445	2,6215	0,1625
105,25	2,1411	1,5489	1,4559	1,4732	2,6719	0,1569
104,33	2,4385	1,6952	1,5851	1,6042	2,7539	0,1482
103,41	2,6342	1,8411	1,7122	1,7372	2,6215	0,1625
102,48	2,7006	1,9536	1,8364	1,8582	2,6719	0,1569
101,55	3,0137	2,1063	1,9589	1,9875	2,6215	0,1625
100,62	3,1996	2,2483	2,0797	2,1160	2,4960	0,1773
99,67	3,3828	2,3517	2,1988	2,2254	2,7539	0,1482
98,72	3,5635	2,4906	2,3162	2,3501	2,6215	0,1625
97,77	3,6848	2,5872	2,4319	2,4575	2,8219	0,1414
